# Effect of Shock-Variable Environmental Temperature and Humidity Conditions on 3D-Printed Polymers for Tensile Properties

**DOI:** 10.3390/polym16010001

**Published:** 2023-12-19

**Authors:** Marcin Głowacki, Katarzyna Skórczewska, Krzysztof Lewandowski, Piotr Szewczykowski, Adam Mazurkiewicz

**Affiliations:** 1 Faculty of Mechanical Engineering, Bydgoszcz University of Science and Technology, Kaliskiego 7 Street, 85-796 Bydgoszcz, Poland; piotr.szewczykowski@pbs.edu.pl (P.S.); adam.mazurkiewicz@pbs.edu.pl (A.M.); 2 Faculty of Chemical Technology and Engineering, Bydgoszcz University of Science and Technology, Seminaryjna 3 Street, 85-326 Bydgoszcz, Poland; krzysztof.lewandowski@pbs.edu.pl

**Keywords:** environment conditions, 3D-printed polymer, tensile properties, shock test

## Abstract

The article presents the research results on the influence of variable shock conditions, such as temperature and water, thus reflecting shock atmospheric conditions during freezing and thawing, on the properties of samples produced using 3D printing technology from commonly used materials such as ABS, HIPS, PLA, and ASA. Understanding how different environmental conditions affect the quality, reliability, and durability of 3D prints can help to optimize the printing process and provide valuable information about their application possibilities. Tests related to the strength of the materials, such as static tensile testing, Charpy impact testing, and evaluation of structures, were carried out using a scanning electron microscope (SEM). Changes in chemical properties were measured by performing tests such as FTIR and TGA. Variations in chemical properties were measured by performing tests such as FTIR and TGA. One shock cycle lasting 7 days was sufficient to alter the properties of 3D prints, with the extent of changes depending on the material, as summarized in the test results.

## 1. Introduction

Materials used in 3D printing technology are often subjected to external influences, illustrating the strong need to analyze the multiple levels of possible changes that can occur as a result of degradation [1,2]. Studying the effects of changing environmental conditions on the properties of 3D prints covered in this article is important for several reasons. First, studying these effects provides a better understanding of how various factors affect the quality and durability of prints, thus allowing the printing process to be optimized and tailored to specific requirements. Different environmental conditions have a significant impact on the mechanical properties of 3D-printed shapes and the durability of the materials used in this technology. High humidity or water can lead to water absorption, a plasticizing effect, hydrolysis, and degradation of some materials. Cyclic temperature shocks in the negative to the positive range, especially combined with the presence of water that freezes or increases the force of migration into the material, can affect the structure, and, thus, the mechanical properties, of 3D prints [3].

The selection of materials for 3D printing and the purpose of the prints may depend on maintaining the properties of elements under environmental factors. This approach leads to the optimization of the printing process. Considering environmental factors and choosing suitable materials can improve the quality and durability of 3D prints. For example, research on the impact of alternating high humidity, temperature, and freezing on the properties of prints made of ABS, HIPS, PLA, and ASA can provide information regarding the impacts of these factors on the properties of these materials [4]. Examples include testing the impact of alternating defrosting and freezing on resin-coated polymer laminates or wood–plastic composites. In both cases, the properties deteriorated [5,6,7]. Currently, researchers have only studied single degrading conditions, such as elevated temperature [8,9,10], aging by sterilization [11,12,13,14,15], aging in gasoline [16], susceptibility to sea salt [17,18,19,20,21,22], freezing [23,24,25], and exposure to salt and sugar solutions [5,26,27]. Only high temperatures affect the strength of ABS, ASA, and PLA materials in degrading environments. A more extensive description can be found in the cited review articles [28,29].

This article discusses the effects of changing conditions, such as temperature shock and the resulting freezing and thawing of samples after exposure to distilled water, on the properties of 3D prints made from ABS, HIPS, PLA, and ASA materials. Understanding how different materials behave under changing conditions and optimizing the 3D printing process is important, so studying these aspects is essential. Moisture and heat resistance are key characteristics of many materials used in various applications. For the materials used in this experiment, moisture resistance, freezing, and varying temperatures were important as these factors can affect the quality and durability of the prints [30,31]. Moisture can also affect the surface properties of prints, such as the appearance of the outer layer; the increase in roughness; and, thus, the quality of the finish [32]. A higher temperature value can significantly affect material properties. It can cause changes in the material’s structure, which can lead to a loss of strength and flexibility [33,34]. In some cases, high temperatures can also lead to the deformation of prints, affecting surface quality. Freezing can cause damage due to water entering the voids of the porous material, which can result in expansion because of freezing [35].

The main objective of this study was to identify various changes in the structure and strength of materials to determine how shock cycles affect the performance of 3D-printed parts. This work provides basic data for analyzing the response of printed parts to the loading regime and environmental conditions. The remainder of this paper is organized as follows: the subsequent section presents comprehensive information about the materials used, the manufacturing process employed, and a detailed description of the experiment. Subsequently, each of the test methods used is discussed. In Section 3, the obtained results and the observed behavior during strength testing of the materials, evaluation of the structures, and changes related to chemical properties are explained. The fourth chapter includes a summary and conclusive remarks that wrap up the article.

## 2. Materials and Methods

### 2.1. Materials and Printing Procedures

The materials used in the study are available from Spectrum’s commercial distribution (PL). HIPS-X has a density of 1.05 g/cm^3^ and the symbol 5903175658012, and is gypsum-white. Smart ABS has a density of 1.05 g/cm^3^ and the symbol 5903175658173, and is polar white. ASA 275 has a density of 1.08 g/cm^3^ and the symbol 5903175653086, and is polar white. PLA Premium has a density of 1.24 g/cm³ and the symbol 5903175657114, and is polar white [36]. A detailed summary of print and platform temperatures is provided in Table 1.

A Zortrax M200 Plus printer (Zortrax S.A., Olsztyn, Poland) was used for printing. Table 2 shows the parameters of the printing process.

**Figure 1:**
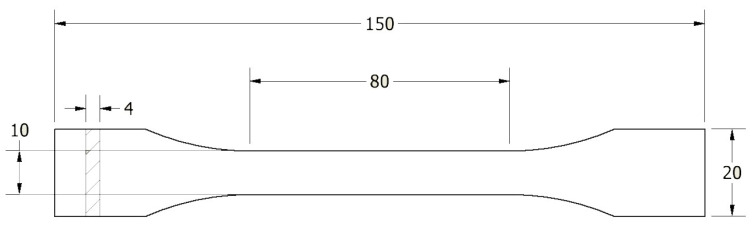
Dimensional projection on a specimen designed according to EN ISO 527:1998 [37].

The dimensions of the specimens designed for tensile properties are shown in Figure 1.

### 2.2. Shock-Variable Environmental Condition Process

Exposure to shock-variable environmental conditions was carried out based on the change in temperature and the presence of water. Samples of the test materials were placed in distilled water for 72 h. Then, after removal from the water, they were placed in a −20 °C freezer for 24 h. Then, the samples were transferred to a 70 °C dryer for 72 h immediately after removal from the freezer. The described procedure cycle was called shock, which was performed three times, and from each stage, i.e., after the 1st, 2nd, and 3rd shock cycles, samples were taken for testing. Selected samples, after measurement of the tensile properties, were subjected to analysis of both their internal and external structures using scanning apparatuses. At the same time, tests were carried out on materials that had not been subjected to shock conditions, which made up the reference samples.

### 2.3. Material Examination

#### 2.3.1. Mechanical Testing

Tensile tests were carried out after each shock cycle and for untreated samples. For static tensile testing, a Zwick Roell Z010 tensile testing machine (Zwick GmbH & Co., KG, Ulm, Germany) was used. The test parameters were as follows: pretension force: 0.1 MPa; tensile speed in the modulus determination area: 1 mm/min; and test speed: 10 mm/min. At least 5 samples in a series were tested each time [37]. Table 3 designates the tensile modulus (*E_t_*), tensile strength (*σ_M_*), elongation at maximum load (*ε_M_*), and strain at break (*ε_B_*).

Charpy impact tests were also performed. Specimens for testing were prepared by cutting a narrow section of the sample, measuring 80 mm × 10 mm × 4 mm [38]. The impact toughness measurement was performed for rectangular samples without notches using the Charpy method by EN ISO 179-1 [38]. Each time, a minimum of 5 samples were tested in a series.

#### 2.3.2. Assessment of Print Structure by Scanning Microscopy

To evaluate the effects of environmental factors on the samples, a JSM-6480LV (JEOL, Tokyo, Japan) scanning electron microscope (SEM) was employed to investigate the surface structure and fractures. Before testing, the samples were sputter-coated with a platinum layer before SEM analysis. Fractured surfaces after static tensile testing were examined at a 1 kV acceleration voltage and at a magnification not exceeding 300×.

#### 2.3.3. Evaluation of Thermal Stability of Prints

To evaluate the thermal stability of materials exposed to environmental conditions in the designed experiment, thermo-gravimetric (TGA) tests were carried out using a TG 209 F3 apparatus from Netzsch Group (Selb, Germany). The measurement was carried out in the temperature range of 30–900 °C in a nitrogen atmosphere at a temperature build-up rate of 10 °C/min. A sample (approx. 10 mg) was taken from the 3D printing samples. The temperature of thermal stability was determined as the temperature at which a 5% weight loss of the material was observed (*T*_5_). The temperatures at which 1%, 10%, and 50% weight losses occurred in the sample were also determined (*T*_1_, *T*_10_, *T*_50_). The temperature at which the decomposition with the highest intensity occurred was determined (*T_DTG_*) from the DTG curves. Based on the obtained thermograms, characteristic values were determined, which are summarized in Table 4. Measurements were performed with two repetitions for each material after the 1st, 2nd, and 3rd shock cycles.

The changes occurring in the material subjected to shock factors were also investigated by Fourier-transform infrared spectroscopy using the Alpha apparatus of the Bruker company and the ATR (reflective) technique. The measurement was conducted in the range of 4400–200 cm^−1^, and 32 scans at a resolution of 4 cm^−1^ were applied.

#### 2.3.4. Statistical Analysis

Based on the statistical analysis, statistical differences between the mean values of the obtained results describing the mechanical properties were assessed. Origin 8.6 Pro software with implemented statistical analysis modules was used for the statistical analysis of the obtained results. ANOVA, with the post hoc Tukey test, was used to compare the significantly different mean values. The normal distribution was confirmed using the Shapiro–Wilk test, and homogeneity of variance was confirmed using Levene’s test. All analyses were performed assuming a significance level below 0.05.

## 3. Results

This chapter will present the results obtained by testing the properties of 3D-printed samples exposed to environmental factors at each of the three stages of shock testing.

### 3.1. Results of Tensile Test

**Figure 2:**
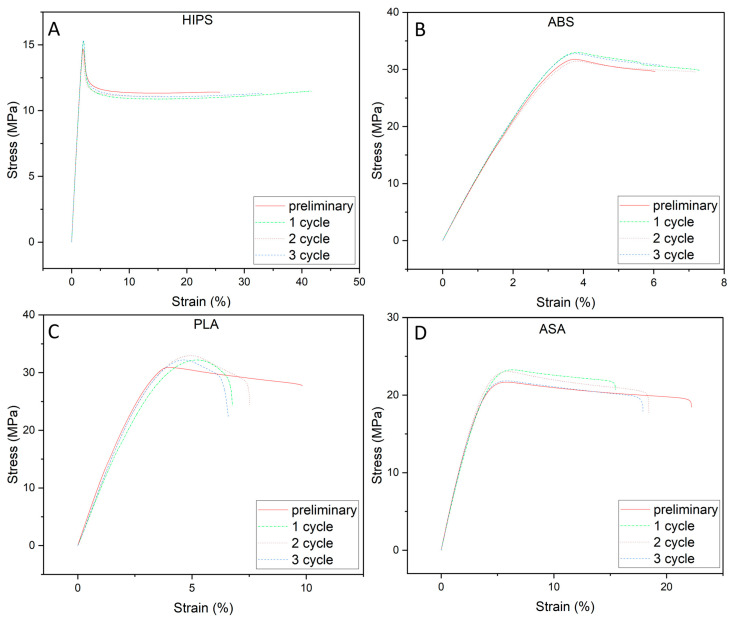
Example stress–strain curves of samples not subjected to environmental factors and samples after each stage of the shock cycle ((**A**)—HIPS, (**B**)—ABS, (**C**)—PLA, (**D**)—ASA).

Figure 2 shows the stress–strain curve obtained during the tensile properties tests.

For specimens made from HIPS, ABS, and ASA, the stress–strain curves were similar, regardless of the number of shock cycles. In contrast, a clear difference can be seen with PLA. The presented curves show only sample specimens from the individual test series. To carry out a broader comparison with a basis for statistical analysis, a number of properties were determined from the stress–strain curves, which are summarized in Table 3. For HIPS and ABS, no significant effect of successive shock cycles on any of the analyzed mechanical properties was found. Based on the analysis performed, significant differences in mechanical properties (tension properties) were found for PLA and ASA. For ASA, there was a slight change in the deformation at the tensile strength, although this was only found for the 1st cycle. For the second and third cycles, the results were the same compared to the reference samples. Here, the change in properties was not to be expected, but rather a measurement inaccuracy. A clear tendency of the change in mechanical properties with shock cycling was found for PLA. A slight increase in tensile strength was observed, with a simultaneous decrease in strain at break. Although no changes were observed for the modulus of elasticity, this may indicate a change in the crystalline structure of the material. The increased temperature applied to PLA may, after increasing the amount of crystalline phase, thus affect the increase in the value of mechanical properties [39].

From the results presented above, it can be concluded that with the HIPS material, the shocks did not cause significant changes to the maximum mechanical strength, but there was a slight decrease in the mean values of the elastic modulus between the initial test and the third cycle. The most significant change occurred in the mean value of strain at break, as it increased by 5.5% relative to the zero test. The ABS material increased its elastic modulus for the samples after the 3rd cycle of being subjected to shocks, reflecting atmospheric conditions. There was also an increase in the stress at break, reducing the strain by 0.7%. In contrast, the PLA material, which belongs to the category of biodegradable materials under the influence of temperature and humidity differences, underwent negative changes in elastic modulus (240 MPa). There were also negative changes in breaking stress and strain at the break by as much as 2.3%. In the case of the ASA material, we observed a minimal decrease in elastic modulus; the stress at break was identical to the initial test. In contrast, the rest of the observed changes were to the disadvantage of the shock-treated samples.

ABS showed a slight increase in stiffness. Heating below the glass transition temperature can cause stress relaxation of the ABS material and result in a restructuring of the macromolecules, increasing the toughness of the joint. This results in a slight increase in stiffness, visible as an increase in the elastic modulus value. The reorganization of the ABS macromolecular segments also increases the mechanical strength of the macromolecules, resulting in a slight increase in *σ_M_* [40]. In addition, the reorganization of the ABS structure into a more ordered one increases the resistance of the material to environmental factors such as water, thus reducing its impact.

### 3.2. Charpy Impact Test

**Figure 3:**
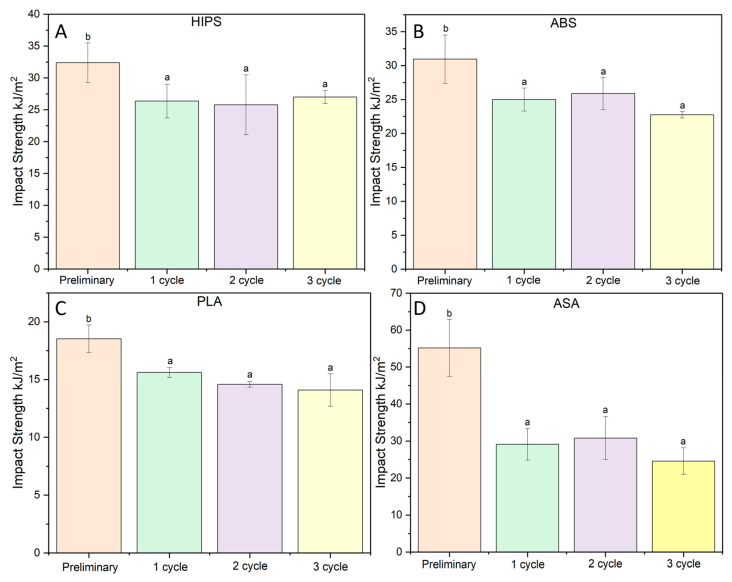
Summary of the Charpy impact strength test results of the samples. Letters (a,b) stand for homogeneous groups.

The summarized impact test results for the reference sample and the samples after each cycle are shown in Figure 3.

The results are shown graphically, with the standard deviation included. The HIPS (A) specimens had an impact strength of 31.6 kJ/m^2^. In just one cycle, the shocked specimens exhibited a 14.5% reduction in impact strength compared to the original sample. Further shock cycles did not result in further changes in the impact strength value, which oscillated around 27 kJ/m^2^. In the case of ABS (B), the impact strength of the blank was 29.7 kJ/m^2^. Here, too, a reduction in this value was observed due to the shock treatment of the samples. Thus, the 1st and 2nd shock cycles caused decreases in impact strength of approx. 15.8%, and the impact strength of the samples after the 3rd cycle was 23.5% lower than that of the original samples.

The impact strength of the PLA samples subjected to environmental shock also decreased by 13.7% for cycle 1. Samples after cycles 2 and 3 were characterized by a similar change in impact strength of approximately 20% compared to the virgin material.

The greatest changes in impact strength were recorded for the ASA samples. A reduction in impact strength of approx. 43% occurred after the first cycle of environmental shock. The second cycle resulted in a slight increase in impact strength, and in the case of the samples, after the third cycle of environmental shock, their impact strength decreased by about 52%.

For all materials, a significant variation in impact strength occurred only after the first cycle. Each subsequent cycle changed nothing; thus, the reduction in the impact strength of the samples after only 1 cycle may have been due to structural changes caused by shock changes in the temperature of the water-exposed samples. This may cause water to migrate through the relatively porous structure of the samples obtained by 3D printing. This, in the subsequent freezing stage, can cause the existing pores to increase in volume, resulting in a weakening of the melting points of the individual filament fibers and a final reduction in impact strength [41,42].

In addition, during the impact test, there was a sharp increase in the stresses distributed unevenly in the sample, which may have resulted in a greater impact on the breaking of bonds between the individual layers of the print and a reduction in the ability to transfer stresses to further filament fibers. The bonds between the individual print layers after one impact cycle were weakened not only inside the fill, but also on the print wall, where these instances of weakening can act as micro-cavities, increasing crack propagation and, thus, reducing impact strength.

### 3.3. Evaluation of the Surface Structure of Samples Using the Scanning Electron Microscope

**Figure 4:**
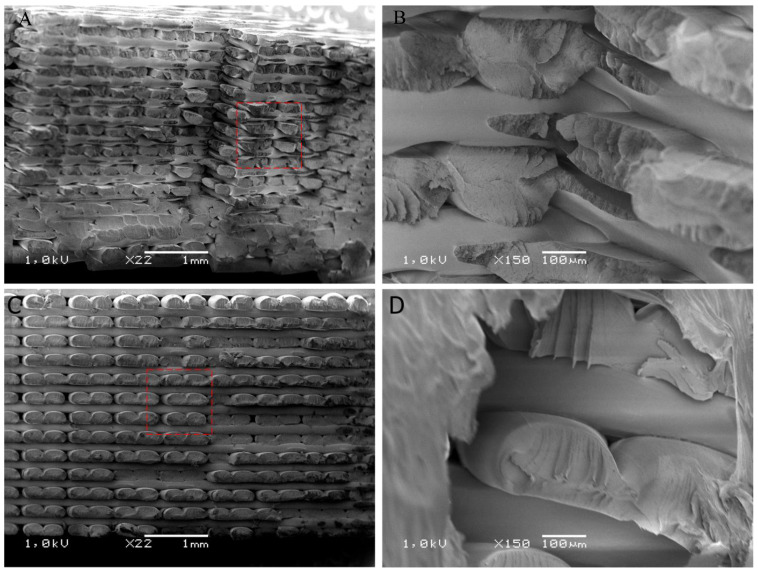
SEM images of the fracture surfaced of the ABS pre-samples (**A**,**B**) and post-surface images after exposure to 3 cycles of shocks (**C**,**D**).

**Figure 5:**
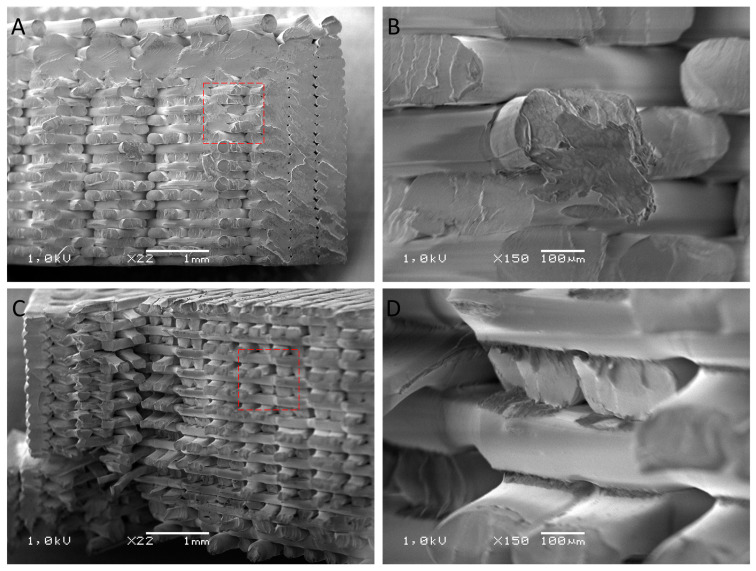
SEM images of the fractured surface of PLA reference samples (**A**,**B**) and post-surface images after exposure to 3 cycles of shocks (**C**,**D**).

**Figure 6:**
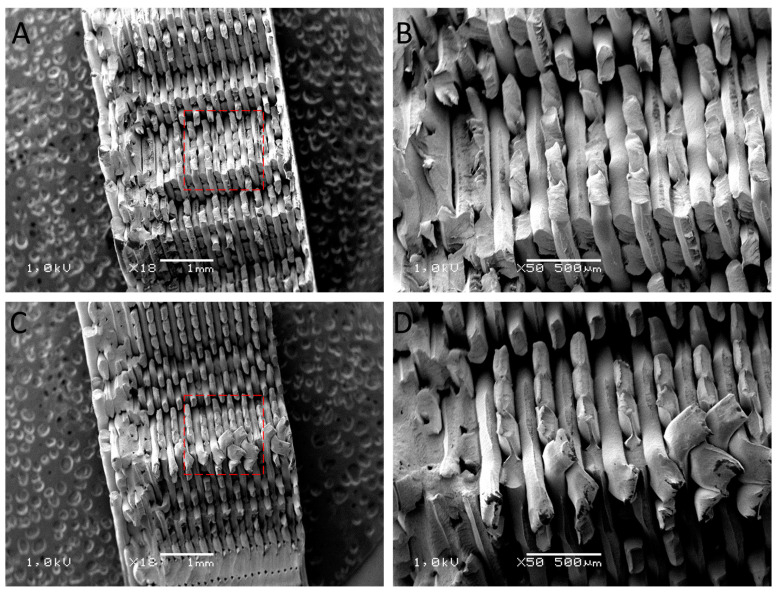
SEM images of the fractured surfaces of the reference samples with ASA (**A**,**B**) and images of the fractured surfaces of the samples after exposure to 3 shock cycles (**C**,**D**).

**Figure 7:**
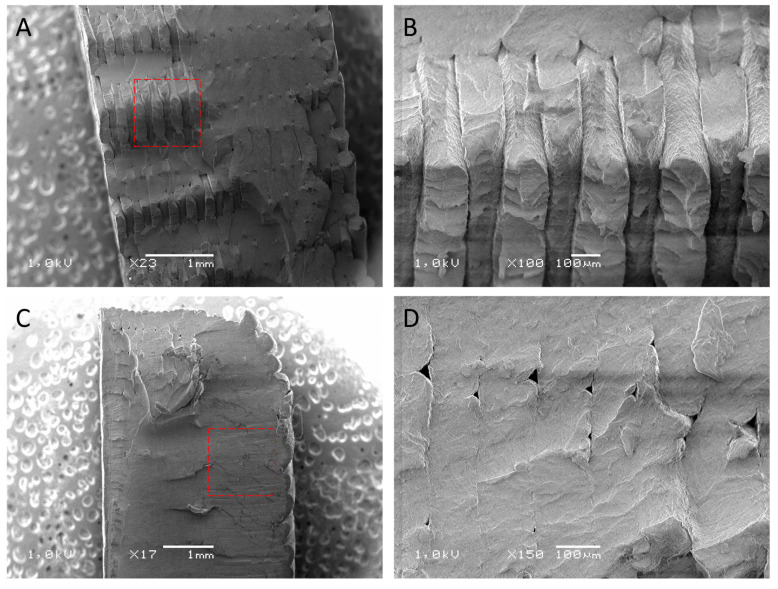
SEM images of the fractured surfaces of the HIPS reference specimens (**A**,**B**) and images of the fractured surfaces of the specimens after exposure to 3 shock cycles (**C**,**D**).

After the mechanical property tests, the samples were used for structural studies. For this, their fracture surface was analyzed. The SEM images (Figure 4, Figure 5, Figure 6 and Figure 7) show images of the fractured surfaces of the untreated samples and of the surfaces of the samples after the 3rd shock cycle.

The structure of the ABS material shaper (Figure 4) did not exhibit any changes caused by the shock effects of varying environmental conditions; in both cases, individual filaments were visible. On this basis, it can be concluded that the applied shock cycles did not affect the structure of ABS specimens, which was reflected in the lack of significant differences in mechanical properties in static stretching. In addition, no differences were observed at the filament bonding points, which indirectly confirms the lack of significant differences in the impact results.

The structure of the print in the case of PLA changed under the influence of the applied shocks (Figure 5). Namely, increased delamination of individual filament fibers was visible in the case of the sample after the 3rd cycle compared to the original material. The layers of filament fibers were clearly separated and less cohesive than in the case of the original sample. In addition, it was observed that the cracking of the individual filaments of the print after the shocks occurred outside the bonding areas (i.e., outside the melting areas of the filament), and in the case of the reference samples, the place of cracking was closer to the bonding surface, i.e., in the areas of immobilization. In addition, in Figure 5C,D, differences like the bonding of individual filament fibers can be observed, which may indicate a weakening of the bonding structure at this location and an increase in the possibility of pulling out the fiber. It can be concluded that the applied shock cycles, which interfered with the structure of the specimen produced via 3D printing, could affect its structure-dependent properties.

Based on the analysis of SEM images of the structure of the PLA samples, it can be concluded that there was delamination of the filament layers caused by exposure to shock changes in environmental conditions in the presence of water. An increase in the presence of gaps between superimposed layers and a decrease in interlayer bonds could translate into a decrease in the mechanical properties of materials exposed to environmental factors through shock tests.

The structure of ASA (Figure 6) showed minimal changes as a result of the shocks, which is noticeable in the SEM images. Microstructural analysis showed insignificant modifications to the structure of the material. The smoothing of individual layers and microcracks was noticeable, which affected the overall strength of the material.

The structure of HIPS changed under the influence of the applied shocks. On the fractured surface of the original sample, the individual layers of the filament could be distinguished, which coherently adhered to each other and were flattened after deposition, giving them a characteristic longitudinal shape. The joint surface was consistent and homogeneous. There were also few free spaces and gaps. The SEM image is characteristic of a brittle fracture with no visible stretching or plastic changes in individual filament fibers. In contrast, the fracture surface of the sample after the 3rd shock cycle was almost smooth, and the structure was almost devoid of spaces between successive layers. It was very difficult to distinguish the individual layers of the filament (Figure 7D).

### 3.4. Analysis of FTIR Results

The spectrum of the samples made of PLA is characteristic of this material. The bands present at the wavelength correspond to CO at 1750 cm^−1^, CH_3_ at 1454 cm^−1^, CH_3_ at 1382 cm^−1^, (CH) at 1360 cm^−1^, CH at 1290–1306 cm^−1^, and COC at 1210 cm^−1^, respectively [43,44].

**Figure 8:**
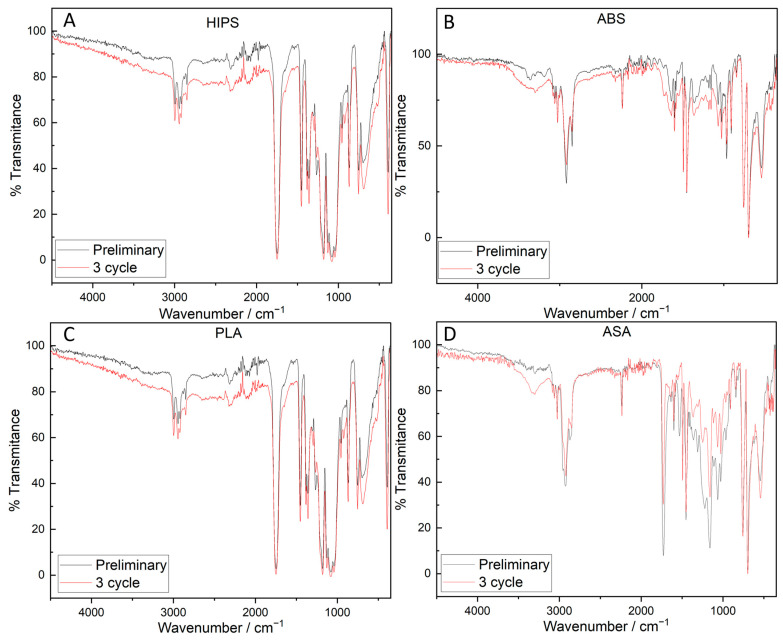
FTIR absorption spectra for HIPS (**A**), ABS (**B**), PLA (**C**), and ASA (**D**).

The FTIR spectrum of the shock-treated sample after the 3rd cycle differed slightly in its course from that of the original material (Figure 8). On the FTIR spectra of the samples after the 3rd cycle, it was observed that there was a decrease in the peak at a wavelength of 1263 cm^−1^ and an increase to 1293 cm^−1^, which suggests the degradation of ketone groups and the appearance of vinyl groups [45,46]. The observed vinyl groups likely came from the formation of ketone groups in the CO-CH=CH_2_ structure [47]. The post-appearance of a peak on the FTIR spectrum of the PLA material after the 3rd cycle, at a wavelength of about 920 cm^−1^, may be associated with ordered regions of α crystals, while the peaks at 957 cm^−1^ correspond to amorphous regions of PLA. A decrease in the intensity of this peak is associated with hydrolytic degradation of amorphous (more susceptible) regions. At the same time, this caused an increase in the degree of crystallinity with the progress of degradation, which is visible as a peak at 921 cm^−1^ [48]. Despite the slight change in the ordering of macromolecules found by FTIR, there was no increase in the material’s stiffness according to mechanical property tests, which may suggest that the reduction in *E_t_* was due not to chemical changes, but to structural changes in individual 3D printing layers formed after the shock cycles.

On the FTIR spectrum of ASA, there were characteristic peaks at about 2240 cm^−1^ associated with the C≡N group; at about 1728 cm^−1^ associated with the presence of C=O; and at 1603 cm^−1^, 1494 cm^−1^, and 1452 cm^−1^ associated with the presence of the benzene ring skeleton. Peaks at 760 cm^−1^ and 699 cm^−1^ represent C-H bending [49].

The spectrum of the sample after the 3rd shock cycle was further characterized by a broad band at 3300 cm^−1^, indicating the presence of OH groups. In addition, a reduction in peak height at 1728 cm^−1^ and in aromatic groups attached to carbonyl groups (1590 cm^−1^) was observed, which may be related to the initiation of thermal degradation of the material [50].

The FTIR spectra of untreated and post-third-shock-cycle ABS samples are typical of ABS. Both spectra show bands at 2230 cm^−1^ ascribed to the -CºN in acrylonitrile; at 1601–1595 and 1452 cm^−1^, ascribed to the aromatic styrene; and at 963 and 910 cm^−1^, related to the butadiene component [51,52]. Similarly, however, there were differences in the intensities of certain bands, suggesting changes in the chemical structure due to environmental factors.

On the spectrum after the 3rd cycle, slight changes in the intensity of individual bands were observed, namely, a more intense, broad peak at about 3300 cm^−1^ related to the presence of OH groups. In addition, peaks at 2850–2930 cm^−1^, ascribed to C-H in the aliphatic segments of ABS, and at 3057 cm^−1^–3047 cm^−1^, related to the C-H in the aromatic benzene rings, showed lower intensity. In addition, a slight increase in the band’s intensity at about 1720 cm^−1^ was associated with an increase in the amount of carbonyl, which may have resulted from the degradation of the polybutadiene rubber phase of ABS material [53]. The observed slight changes in the chemical structure may indicate degradation initiated by environmental conditions, or may be related to an increase in the sample’s roughness surface and a slight shift in the analyzed spectra obtained via the ATR technique.

A similar nature to that of the changes to ABS was observed by analyzing the FTIR spectra of samples made from virgin HIPS which were exposed to shock changes in environmental conditions. Again, slight changes were observed in the hydroxyl region (between 3600 and 3100 cm^−1^), the carbonyl region (between 1800 cm^−1^ and 1620 cm^−1^), and the double bond region (between 1000 and 880 cm^−1^) [54]; these were related to the effects of environmental factors on the sample material.

### 3.5. Analysis of TGA Results

**Figure 9:**
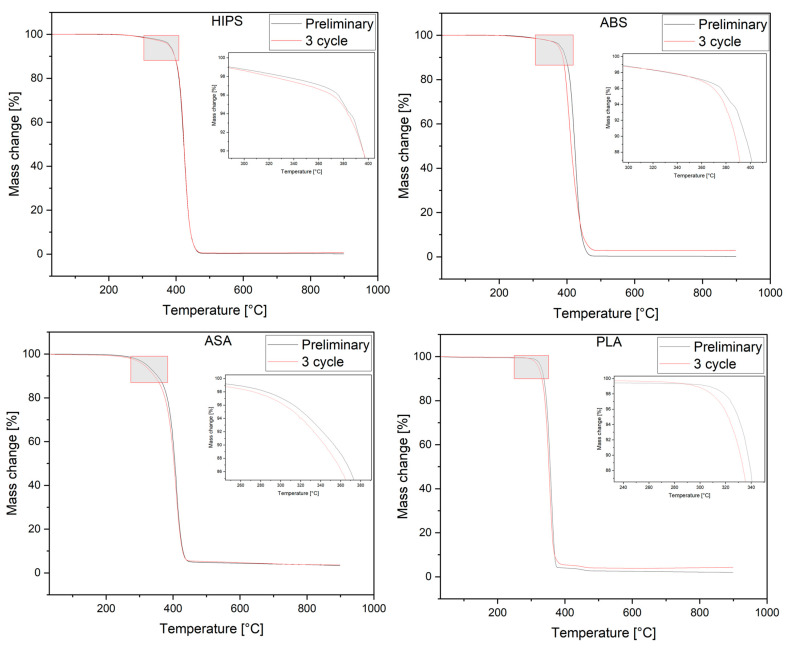
TGA plots of samples, both untreated and after 3 shock cycle.

Figure 9 illustrates the thermogram progression for each material type in the original sample, both untreated and after three shock cycles. The TGA thermograms of the tested materials, irrespective of the environmental action, are typical for these polymers, i.e., decomposition occurred in a single step, and only in the case of PLA was an additional step related to the presence of a mineral color modifier compound observed [35].

However, with PLA and ASA, a reduction in thermal stability was observed with the shock cycle to which the specimens were subjected. This was evidenced by a decrease in the temperature values at which 1%, 5%, and 10% of the sample weight was lost (*T*_1_, *T*_5_, *T*_10_) These changes show the degrading effect of the shock conditions on the tested samples. In the case of PLA, the change in *T*_1_ was 10.6 °C, and that in *T*_5_ was 5.3 °C.

ASA degradation proceeded in a single step. These results are in agreement with the literature [55]. The material was thermally stable up to a temperature of about *T*_5_ 321.7 °C, at which point there was a 5% weight loss of the sample, and the T_DTG_ degradation maximum occurred at 409.5 °C. The ASA materials after the 1st and 2nd shock cycles were characterized by a slightly reduced *T*_5_ value of about 2.5 °C, and in the case of the 3rd cycle, there was a reduction in this value by 10 °C. Also, pronounced changes in the value of *T*_10_ could be observed in samples after the 3rd shock cycle. On the other hand, the values of *T*_50_, *T_onset_*, and *T_DTG_* were comparable regardless of the shock cycle, indicating that the effects associated with the environmental factors were evident at the initial stage of TGA testing.

By analyzing the TGA thermograms (Figure 9) and the values in Table 4 of the HIPS and ABS materials, one can conclude that there was a significant reduction in thermal stability under the applied shock conditions. The *T*_1_ and *T*_5_ temperature changes in HIPS and ABS after the 3rd shock cycle compared to the original material were 0.9 °C and 0.2 °C and 3.4 °C and 0.8 °C, respectively. Through further analysis of the TGA thermograms, we did not observe significant differences between the original and shock-cycled material, which would indicate degradation changes in the material. It can be concluded that the applied environmental factors do not affect the reduction in the thermal stability of these materials, which suggests an absence of changes in the chemical structure of the materials.

The applied shock cycles, in the case of PLA, caused the greatest changes among the tested materials, which is in line with the properties of this polymer. Namely, the material showed reduced resistance to water, which can cause hydrolytic degradation [56]. An additional factor was elevated temperature, which can also intensify oxidative degradation processes that reduce the thermal stability of the material. According to the literature [55], the hydrolysis of ester bonds of PLA macromolecules in the presence of moisture, especially amorphous regions, can lead to the separation of oligomers, with the effect of faster degradation of the material and lower thermal stability. Nevertheless, the observed reduction in thermal stability does not affect the serviceability of these materials, the temperature range of which is considerably lower; it only signals some initiation of degradation processes associated with reduced resistance to the applied shock conditions.

## 4. Summary and Conclusions

Environmental conditions were simulated on the water-treated specimens as shocks due to changes in temperature from negative to positive. Conventional materials commonly used in 3D printing, such as PLA, ABS, HIPS, and ASA, were selected for the study. It is important to note that plastics such as ASA, HIPS, and ABS have relatively good resistance to weathering and elevated temperatures and moisture. An interesting aspect, however, was the behavior of the 3D prints, which differed fundamentally in structure from the solid materials.

Just one shock cycle of 7 days (1 day soaking in distilled water, 3 days at −20 °C, and 3 days at 70 °C) resulted in changes in the properties of the 3D prints, varying according to the material used.

Based on the results of the static tensile mechanical properties tests conducted on the HIPS, ABS, and ASA prints, no significant changes in these properties were observed depending on the shock cycles used. However, in the case of PLA samples, a decrease in Et values was observed. Interestingly, a significant change in the impact strength of the prints occurred after just one impact cycle regardless of the type of filament used. These changes were due to the change in the structure of the conditioned samples and the arrangement of the individual layers of the filament after the impact cycle [57].

The structure of the 3D prints changed depending on the filament material after applying cyclic shocks to environmental conditions. The most significant changes occurred with 3D shapes made of PLA and HIPS, with PLA experiencing increased delamination of its filament layers, while HIPS showed a reduction in interlayer pores and even melting of the filament fibers. The structure of the 3D shapes made from ABS and ASA did not change significantly after exposure.

Analysis of the FTIR results showed changes in the chemical structure of the sample material. Insignificant changes were observed for HIPS and ABS, while for PLA, changes in the chemical structure related to hydrolytic degradation and an increase in the degree of crystallinity, which could also result from initial hydrolytic degradation of the material, were observed. The TGA results also confirmed the initiation of degradation in the case of PLA as the number of cycles increased, as well as slight changes in thermal stability in the cases of HIPS and ABS materials, irrespective of the shock cycles. In contrast, for ASA, significant changes in thermal stability were only observed after the 3rd shock cycle.

The applied shock-variable environmental conditions can synergistically influence structural changes in the 3D specimen and chemical structure of the material, depending on its type. In addition, the presence of water can cause it to migrate inwards through the interlaminar gaps in the filament, leading to its subsequent shock freezing and a change in its volume, thus causing damage to the interfaces of the filament and weakening the structure of the print.

## Figures and Tables

**Table 1:** Print temperatures.

Material	Extrusion Temperature	Platform Temperature
Smart ABS	275 °C	80 °C
HIPS-X	250 °C	80 °C
ASA 275	240 °C	60 °C
PLA Premium	210 °C	30 °C

**Table 2:** FDM process parameters used for sample preparation.

Parameter Name	Parameter Value
Infill pattern	Rectilinear
Fill angle	45°
Infill density	100%
Layer height	0.3 mm
Solid layers	Top 7, Bottom 4
Print speed	30 mm/s
nozzle diameter	0.4 mm
diameter tolerance	+/−0.03
Layer height	0.19 mm
Filling density	100%

**Table 3:** Summary of results of static tensile mechanical properties tests.

	HIPS				ABS				PLA				ASA			
	0	1	2	3	0	1	2	3	0	1	2	3	0	1	2	3
*E_t_* (MPa)	851 ^a^ ±15.0	846 ^a^ ±17.8	852 ^a^ ±20.7	843 ^a^ ±19.9	1140 ^a^ ±34.6	1170 ^a^ ±5.65	1150 ^a^ ±47.2	1180 ^a^ ±12.9	1160 ^a^ ±2.66	934 ^a^ ±80.4	1010 ^a^ ±122	914 ^a^ ±168	673 ^a^ ±12.6	643 ^a^ ±31.5	649 ^a^ ±8.61	645 ^a^ ±8.02
*σ_M_* (MPa)	14.8 ^a^ ±0.69	15.1 ^a^ ±0.44	14.6 ^a^ ±0.51	15.1 ^a^ ±0.26	31.9 ^a^ ±1.22	33.1 ^a^ ±0.78	32.1 ^a^ ±1.67	33 ^a^ ±0.72	31.2 ^a^ ±0.57	32.2 ^a,b^ ±0.23	32.4 ^b^ ±0.36	32.3 ^b^ ±0.27	21.9 ^a^ ±0.6	22.8 ^a^ ±1.93	22.5 ^a^ ±0.48	21.9 ^a^ ±0.3
*ε_M_* (%)	2 ^a^ ±0	2 ^a^ ±0	2 ^a^ ±0	2.1 ^a^ ±0	3.8 ^a^ ±0.1	3.8 ^a^ ±0.1	3.8 ^a^ ±0.1	3.7 ^a^ ±0.1	4.1 ^a^ ±0.1	5.1 ^b^ ±0.1	4.7 ^b^ ±0.2	4.7 ^b^ ±0.2	5.8 ^a^ ±0	6.2 ^b^ ±0.2	5.8 ^a^ ±0.1	5.7 ^a^ ±0.1
*ε_B_* (%)	29.7 ^a^ ±6.3	33.8 ^a^ ±6.5	24.2 ^a^ ±10.2	35.2 ^a^ ±5.0	6.3 ^a^ ±1.2	6.3 ^a^ ±1.3	6.7 ^a^ ±1.6	5.6 ^a^ ±0.8	8.9 ^c^ ±0.6	8.1 ^b,c^ ±0.8	6.9 ^a,b^ ±0.8	6.6 ^a^ ±0.6	18 ^a^ ±3.6	20.2 ^a^ ±4.8	18.9 ^a^ ±3.6	17.1 ^a^ ±1.3

Index indicates homogeneous groups within a single material. Letters (a,b,c) stand for homogeneous groups.

**Table 4:** The TGA analysis results.

Material		*T*_1_	*T*_5_	*T*_10_	*T*_50_	*T_ONSET_*	*T_DTG_*
	Cycle	(°C)	(°C)	(°C)	(°C)	(°C)	(°C)
PLA	0	306.2	329.8	337.5	356.2	341.6	360.2
	1	301.4	326.3	335.3	356.3	340.3	359.5
	2	297.1	325.7	335.3	356.4	340.4	360.2
	3	294.8	320.2	329.9	350.6	335.3	353.8
HIPS	0	289.9	380.4	395.8	422.9	404.6	425.5
	1	291.7	381.5	395.8	422.8	405.1	424.8
	2	291.1	381.5	397.5	424.0	406.6	426.5
	3	290.8	381.3	396.6	423.8	405.6	424.7
ASA	0	255.7	321.7	353.1	405.6	386.5	410.6
	1	256.1	318.9	350.6	406.0	385.0	409.5
	2	255.3	318.4	348.0	405.5	384.8	410.0
	3	237.4	311.3	343.6	405.4	384.9	411.2
ABS	0	305.8	377.9	390.4	415.5	390.0	412.3
	1	305.6	377.4	389.3	414.8	388.8	410.4
	2	302.0	378.6	390.0	414.8	389.8	408.9
	3	302.4	378.7	389.8	414.9	389.6	408.9

## Data Availability

The data supporting this study’s findings are available from the corresponding authors (Marcin Głowacki and Katarzyna Skórczewska) upon request.
